# An Investigative Analysis of Therapeutic Strategies in Hepatocellular Carcinoma: A Raetrospective Examination of 23 Biopsy-Confirmed Cases Emphasizing the Significance of Histopathological Insights

**DOI:** 10.3390/cancers16101916

**Published:** 2024-05-17

**Authors:** Anca Zgura, Mugur Cristian Grasu, Radu Lucian Dumitru, Letitia Toma, Laura Iliescu, Cosmin Baciu

**Affiliations:** 1 “Carol Davila” University of Medicine and Pharmacy, 050474 Bucharest, Romania; anca.zgura@umfcd.ro (A.Z.); radu.dumitru@umfcd.ro (R.L.D.); letitia.toma@umfcd.ro (L.T.); elena.iliescu@umfcd.ro (L.I.); constantin.baciu@umfcd.ro (C.B.); 2 Department of Interventional Radiology, Fundeni Clinical Institute, 022328 Bucharest, Romania; 3 Department of Internal Medicine, Fundeni Clinical Institute, 022328 Bucharest, Romania

**Keywords:** cancer, hepatocellular, biopsy, alpha-fetoprotein, immune checkpoint inhibitor

## Abstract

**Simple Summary:**

This retrospective study focused on 23 patients diagnosed with Hepatocellular Carcinoma (HCC), utilizing the Liver Imaging Reporting and Data System (LI-RADS) for classification. This system aids in the non-invasive diagnosis of HCC, potentially eliminating the need for a diagnostic biopsy. Patients were selected based on their diagnosis, confirmed either via imaging methods that identified advanced liver nodules or via histopathological findings consistent with HCC, including specific protein markers. The study aimed to assess the histopathological changes resulting from prior local interventions, such as trans-arterial chemoembolization or radiofrequency ablation, and their impact on the tumor’s response to subsequent immune therapies. Key findings indicated variations in alpha-fetoprotein levels and increased expression of the immune marker PD-L1 in untreated patients, suggesting a more aggressive cancer progression in these individuals. The study’s conclusions support the use of liver biopsy in refining therapeutic approaches for HCC, particularly in recurrent cases post-intervention, to enhance personalized immune therapy strategies.

**Abstract:**

Background: The Liver Imaging Reporting and Data System (LI-RADS) combines standardized terminology with a classification system for imaging findings in patients with HCC, therefore rendering diagnostic biopsy unnecessary in many cases. This retrospective study included 23 patients with a biopsy diagnosis of HCC, performed either before or after local interventional procedures, in order to evaluate the histopathologic changes induced by previous procedures and their potential influence on the response to immune therapy. Material and Methods: The study encompassed a cohort of patients diagnosed with Hepatocellular Carcinoma (HCC). Diagnosis was established via contrast-enhanced computer tomography or magnetic resonance imaging that identified LI-RADS-5 nodules in conjunction with historical liver disease and elevated alpha-fetoprotein (AFP) levels or via histological examination confirming positivity for glypican3, heat shock protein 70, and glutamine synthetase. The study detailed the liver disease etiology, LI-RADS scores, characteristics and dimensions of HCC nodules, serum AFP concentrations, Edmondson–Steiner grading, and the expression of programmed cell death ligand 1 (PD-L1) in the tumor cells. Results: Among the study’s cohort of Hepatocellular Carcinoma (HCC) patients, a portion had not received any prior treatments, while the remainder experienced local HCC recurrence following trans-arterial chemoembolization or radiofrequency ablation. Observations indicated elevated alpha-fetoprotein (AFP) levels in those who had not undergone any previous interventions, showing statistical significance. The Edmondson–Steiner classification predominantly identified grade III differentiation across patients, irrespective of their treatment history. Furthermore, an increase in intra-tumoral programmed cell death ligand 1 (PD-L1) expression was noted in patients who had not been subjected to previous therapies. Conclusion: Liver biopsy offers valuable insights for patients with Hepatocellular Carcinoma (HCC), assisting in the tailoring of immune therapy strategies, particularly in cases of recurrence following prior local interventions.

## 1. Background

Hepatocellular Carcinoma (HCC) remains a public health challenge, despite the numerous diagnostic and therapeutic means currently available [1]. In 2018, an estimated 841,000 HCC cases were diagnosed, and 782,000 HCC-associated deaths were recorded [2]. An estimate using the GLOBOCAN database found over 900,000 new HCC cases in 2020 and predicted an incidence increase of 55% to about 1.4 million patients diagnosed in 2040 [3]. Some authors estimate that the incidence of HCC will rise to approximately 1 million patients per year by 2025 [4]. Increasing awareness of chronic viral hepatitis, as well as the high rates of sustained virologic response obtained by direct-acting antiviral therapy for hepatitis C, were expected to lower the incidence of HCC [5]. However, the rising prevalence of obesity and metabolic syndrome leads to an increase in non-alcoholic fatty liver disease and its complications (cirrhosis and HCC), overcoming the benefits of virologic advances [6].

Current guidelines recommend a personalized approach regarding the screening, diagnosis, and management of HCC [5,7]. The European Association for the Study of the Liver (EASL) recommends periodic surveillance for HCC in high-risk patients such as patients with advanced liver disease (patients with cirrhosis) or patients with advanced fibrosis (F3) and hepatitis B infection [5]. The American Association for the Study of Liver Diseases (AASLD) also refers to cirrhosis as the main risk factor for HCC, particularly with underlying viral etiology, but raises awareness of the increasing incidence of metabolic syndrome-associated HCC [7]. According to AASLD guidelines, HCC screening should be performed in patients with Child–Pugh class A or B cirrhosis (as it is not cost-efficient for Class C cirrhosis) and in non-cirrhotic hepatitis B patients [7].

The diagnosis of HCC requires evaluation of risk factors (chronic liver disease), tumoral markers (alpha-fetoprotein (AFP) being the most commonly used), and imaging patterns based on the enhancement after intravenous contrast [5]. EASL guidelines recommend non-invasive tests for HCC diagnosis in patients with cirrhosis, comprising serum markers and imaging, while strongly recommending a histological diagnosis in patients without cirrhosis [5]. Non-invasive criteria should only be applied to patients with cirrhosis with nodules larger than 1 cm. Imagining techniques include multiphasic computer tomography (CT) or dynamic contrast-enhanced magnetic resonance imaging (MRI); contrast-enhanced ultrasonography (CEUS) shows similar specificity and sensitivity but currently has insufficient evidence for a strong recommendation [5,8]. The imagistic hallmark of HCC consists of arterial hyperenhancement with delayed washout, attributable to the vascular abnormalities associated with HCC [9]. Typical MRI aspects include hyperintensity in the arterial phase, hypo-intensity in the portal phase, and hypo-intensity in the transitional phase [10].

Imaging features of liver nodules may be classified according to LI-RADS score, to predict the possibility of malignancy depending mainly on nodule dimensions and enhancement pattern [11]. Importantly, this score also aids in establishing the need for liver biopsy for atypical nodules or nodules with malignancy characteristics and low HCC risk. This classification defines eight categories of liver nodules, according to the CT or MRI aspect, according to the probability of malignancy of a liver nodule [12]:

- LR-NC, non-categorizable due to degraded images;
- LR-1, definitely benign;
- LR-2, probably benign, referring to nodules less than 2 cm, without any imagistic criteria of malignancy;
- LR-3, intermediate probability of malignancy, referring to nodules less than 2 cm with non-rim arterial hyperenhancement or nodules larger than 2 cm with arterial iso or hypo-enhancement;
- LR-4, high HCC probability, referring to nodules less than 10 mm, with arterial hyperenhancement and one other typical feature or nodules over 20 mm with arterial hyperenhancement and no other suggestive features;
- LR-5, a definite diagnosis of HCC, nodules over 10 mm with arterial hyperenhancement and portal washout, or with a 50% size increase in less than 6 months;
- LR-TIV, malignant venous thrombus, with arterial hyperenhancement regardless of the presence of a liver nodule;
- LR-M, high malignancy probability, but not HCC, referring to nodules with rim arterial enhancement, peripheral washout, targetoid aspect, or infiltrative appearance.

LI-RADS criteria have been validated in patients with cirrhosis or patients with severe fibrosis and hepatitis B (HBV) infection; therefore, in all other situations, histology analysis is required for a definite HCC diagnosis [7]. Pathologic diagnosis is based on criteria established by the World Health Organization and the International Consensus Group for Hepatocellular Neoplasia and takes into consideration stromal invasion, increased cell density, intratumoral portal tracts, excessive arteries, a pseudoglandular pattern, and diffuse fatty changes. A panel of 3 immunohistological markers (heat shock protein 70, glypican 3, and glutamine synthetase) has shown good specificity and sensitivity in HCC diagnosis in cases where histology is controversial [13]. Edmondson Steiner grade is a histologic predictor for HCC recurrence, classifying HCC as well differentiated, moderately differentiated, poorly differentiated, and pleiomorphism [14]. A retrospective trial found that high Edmondson–Steiner degrees correlate to high AFP levels, large or infiltrative tumors, and advanced HCC but did not correlate with outcomes after local procedures (chemoembolization, ablation, and yttrium-radioembolization) [14].

The therapeutic decision is based on the Barcelona Clinic Liver Cancer (BCLC) classification, which stratifies patients according to tumor burden, clinical status, and baseline liver function [15]. For patients with very-early-stage and early-stage HCC, curative procedures are recommended (surgical resection or radiofrequency ablation (RFA), liver transplantation) [7]. Patients with intermediate-stage HCC are also candidates for liver transplant but may undergo transarterial chemoembolization (TACE) or systemic therapies, while patients with advanced-stage HCC should receive systemic therapies alone [7]. The 2018 EASL guideline and the 2023 AASLD guidelines have different recommendations for first-line systemic therapies: EASL recommends sorafenib or lenvatinib, while AASLD recommends atezolizumab/bevacizumab or durvalumab/tremelimumab [5,7].

As mentioned before, systemic therapies are currently reserved for patients with advanced disease, disease progression, or patients with nodules amendable to local therapies that are unavailable or technically impossible [16]. These therapies include tyrosine kinase inhibitors (sorafenib, lenvatinib, regorafenib and cabozatinib), an inhibitor of the vascular endothelial growth factor receptor 2 (ramucirumab) and immune checkpoint inhibitors (ICI) (atezolizumab associated with bevacizumab, ipilimumab associated with nivolumab, nivolumab and pembrolizumab as monotherapies) [16].

Recent data suggest that intra-tumoral biomarkers may predict the response to immunotherapy, especially to immune checkpoint inhibitors, currently the first line of systemic therapy [17]. For instance, programmed cell death ligand 1 (PD-L1) expression in tumor cells (associated with poor differentiation and macrovascular invasion) correlates with a better response to immune therapy [18]. As new markers and treatments emerge, it is important to personalize the management of the patients to achieve the best prognosis in the setting of limited curative interventions [17]. As such, tumor biopsy regains importance in HCC management, even in patients with a definite imaging diagnosis. A recent review summarizes predictive biomarkers for HCC prognosis and response to immune therapy, including PD-L1 expression in tumor cells, DNA Damage Repair pathways expression, tumor mutational burden, and tumor-infiltrating lymphocytes [19]. There is controversial evidence regarding PD-L1 as a prognosis marker. Overexpression of PD-L1 in HCC histology has been associated with poor prognosis in a series of 217 HCC patients, but the IMBrave150 trial revealed thatPD-L1 expression predicted a good outcome in patient treated vit atezolizumab/bevacizumab compared with patients treated with sorafenib [20,21].

According to the National Guidelines, atezolizumab/bevacizumab therapy is reserved for patients with Class A cirrhosis, with progression after local therapies or sorafenib therapy with a positive HCC diagnosis established by contrast-enhanced imaging in patients with cirrhosis or by histology in patients without cirrhosis [22]. In this setting, we performed liver biopsies on eligible HCC patients for therapy approval. To address the inquiries regarding the rationale behind performing biopsies, it is pertinent to clarify that biopsies were undertaken based on a comprehensive assessment of each patient’s clinical presentation and diagnostic imaging findings. Biopsies prior to treatment were primarily conducted to confirm diagnoses in complex cases where imaging alone did not provide definitive evidence of HCC, following the EASL and AASLD guidelines. Post-treatment biopsies were performed to evaluate the histopathological effects of treatment and identify any signs of recurrence or residual disease. This approach was guided by the institution’s protocol, which aligns with national guidelines, considering factors such as tumor characteristics, patient’s liver function status, and overall clinical context.

This retrospective study included 23 patients with a biopsy diagnosis of HCC to evaluate whether histological differences before and after local procedures may help predict the response to systemic therapy.

## 2. Materials and Methods

### 2.1. Ethical Statement

All the participants signed informed consent forms for the medical procedures and for participation in medical studies. The study is approved by the Local Ethics Committee (1718/2021).

### 2.2. Patient Selection

Our study entailed a retrospective analysis of 76 liver tumor biopsies conducted at our facility from January 2021 to January 2022. These liver biopsies were executed percutaneously, guided by computer tomography (CT) scans, and carried out by an experienced interventional radiologist. A senior pathologist then evaluated the biopsies for size and histopathological characteristics.

We excluded patients from the study if their imaging indicated liver metastases (classified as LR-M according to LI-RADS criteria), if they had a known history of solid malignancies, or if the biopsy results confirmed liver metastases (total exclusions: 53). In cases where histological findings were ambiguous, we employed an immunochemistry panel to definitively diagnose Hepatocellular Carcinoma (HCC). This process resulted in a subset of 33 biopsies being selected for further examination of PD-L1 expression.

For each participant, we documented the etiology of the underlying liver disease. We categorized HCC patients based on their treatment history: those who had not undergone any previous local therapies, those who experienced recurrence following Transarterial Chemoembolization (TACE), and those with recurrence after Radiofrequency Ablation (RFA). Additionally, we compiled data on serum alpha-fetoprotein (AFP) levels for each patient.

### 2.3. Imaging Techniques

**Figure 1:**
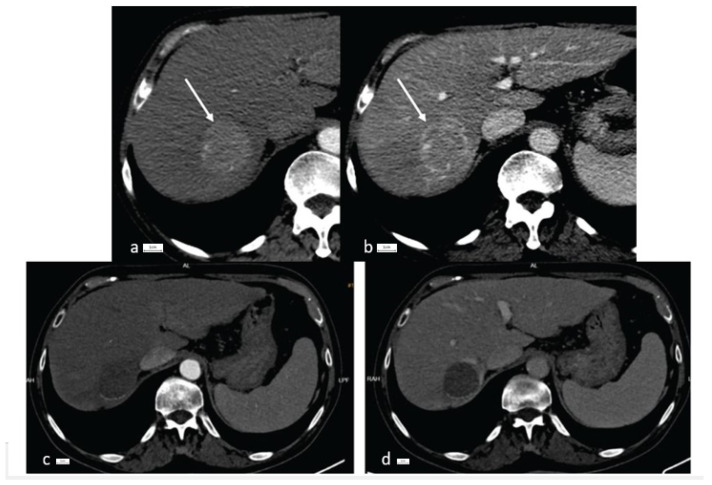
CT imaging of HCC in an 84-year-old patient before and after interventional therapy. Initial contrast-enhanced CT showing a 45/50 mm tumor (arrow) in the 7th segment, with arterial hyperenhancement (**a**) and portal wash-out (**b**). CT imaging after TACE and RFA showing no hyperenhancement in the arterial phase (**c**) and no wash-out in the portal phase (**d**).

Patients underwent contrast-enhanced abdominal CT scan or MRI before liver biopsy. We collected data regarding the size and number of nodules, portal vein invasion, and LI-RADS degree (LR-4 or LR-5 in LI-RADS criteria). All nodules had a diameter of over 10 mm and presented nonrim arterial hyperenhancement (Figure 1).

### 2.4. Histology Analysis

In our cohort, histology was pursued in all patients where non-invasive diagnostic criteria were not fully conclusive or when specific histopathological information was deemed necessary for tailoring treatment strategies. The decision to perform a biopsy was influenced by a multidisciplinary team discussion, taking into account the potential benefits and risks associated with the procedure. Local guidelines at our center recommend biopsy in situations where additional diagnostic clarity could significantly impact patient management decisions, particularly in cases without clear LI-RADS categorization or when atypical radiological features are present.

**Figure 2:**
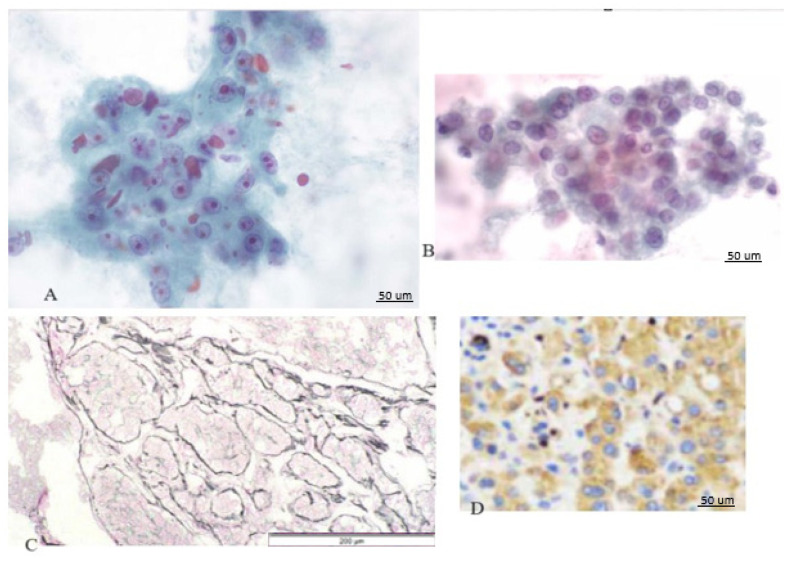
Histology aspects of HCC. (**A**) increased N/C ratio, macronucleoli, and hyaline globules (hematoxylin-eosin stain ×400). (**B**) Cellular monomorphism suggestive of malignancy (hematoxylin-eosin stain ×400). (**C**) Trabecular pattern (hematoxylin-eosin stain ×20). (**D**) PD-L1 overexpression in HCC cells.

Following the collection of samples, the tissue underwent histological examination. The identification of Hepatocellular Carcinoma (HCC) relied on specific characteristics, including polygonal cells exhibiting nuclear irregularities, an elevated nucleus-to-cytoplasm ratio, pronounced nuclei, uneven nuclear contours, and the presence of multiple nuclei. Additionally, the analysis included the observation of a trabecular pattern (refer to Figure 2).

In patients without a clear histology diagnosis, we performed a panel of immunohistochemical testing consisting of glypican3, heat shock protein70, and glutamine synthetase. After a definitive HCC diagnosis, we determined the expression of PD-L1 in tumor cells by immunohistochemistry on formalin-fixed paraffin-embedded sections.

### 2.5. Statistical Analysis

Statistical analysis was performed using SPSS (IBM Corp. Released 2019. IBM SPSS Statistics for Windows, Version 26.0. IBM Corp.: Armonk, NY, USA). Numerical data such as AFP levels and PD-L1 expression are described as means. Qualitative variables are described as percentages. Correlations between AFP levels or PD-L1 expression and previous interventional therapies were determined using a chi-square test.

## 3. Results

### 3.1. Descriptive Data

This research encompassed 23 participants, comprising 12 men and 11 women, with an average age of 51.04 years, plus or minus 13.2 years. The classification of patients was based on prior treatments administered to the same region as the biopsied nodule, categorizing them into groups of no prior intervention, those who underwent Radiofrequency Ablation (RFA), or those treated with Transarterial Chemoembolization (TACE). Individuals whose biopsies were taken from newly identified nodules were categorized as having received no previous interventions. Detailed demographic information and patient data are summarized in Table 1.

Consistent with expectations, a predominant number of lesions were identified in patients with chronic viral hepatitis. Specifically, an analysis revealed that 8 of the 9 patients infected with the Hepatitis C Virus (HCV) had received antiviral therapy, resulting in a sustained virologic response. In the cohort of patients with Hepatitis B Virus (HBV) infection, 3 out of 8 were being treated with nucleoside analogs. Furthermore, a significant difference in alpha-fetoprotein (AFP) levels was observed, with higher AFP levels noted in patients who had not received any prior interventions on the lesion of interest in comparison to those who had undergone prior interventions (*p* = 0.02).

### 3.2. Liver Biopsy Outcomes

For three patients who had undergone Transarterial Chemoembolization (TACE) and one patient previously treated with Radiofrequency Ablation (RFA), liver biopsies were repeated due to initial inconclusive outcomes. It is noteworthy that none of the patients experienced complications post-biopsy.

The dimensions of the biopsy samples were approximately 1.2 cm (ranging from 0.7 to 1.5 cm) in patients without prior treatments, 1.1 cm (ranging from 0.6 to 1.3 cm) in those previously subjected to TACE, and 1.2 cm (ranging from 0.8 to 1.4 cm) in the cohort with prior RFA treatments. Histopathological examination of all biopsy specimens confirmed the diagnosis of Hepatocellular Carcinoma, predominantly demonstrating moderate differentiation as per the Edmondson-Steiner grading system (refer to Table 2).

Additionally, the examination of programmed death-ligand 1 (PD-L1) expression indicated elevated levels in patients who had not undergone any previous treatments, although this difference did not reach statistical significance (*p* = 0.6).

In evaluating the efficacy of the therapeutic intervention, we considered not only the overall burden of disease but also the specific biological mechanisms that may predict response to treatment. To this end, we quantified the expression of PD-L1 using two distinct but complementary indices: the Combined Positive Score (CPS) and the Tumor Proportion Score (TPS). The TPS metric, which measures the percentage of viable tumor cells showing partial or complete membrane staining of PD-L1, provides insight into the extent of PD-L1 expression within the tumor microenvironment. TPS values ranged from 8.7% to 11.6%, indicating a moderate level of PD-L1 expression in tumor cells. On the other hand, the CPS takes into account PD-L1 positivity among all cells within the tumor microenvironment, including tumor cells, lymphocytes, and macrophages. This score is particularly useful in assessing the broader immunological landscape and may be reflective of both innate and adaptive immune responses. The comparative analysis of CPS and TPS before and after treatment revealed insightful trends. Pre-treatment tissues displayed a certain heterogeneity in PD-L1 expression, with CPS and TPS providing distinct perspectives on the immunogenicity of the tumors. Post-treatment samples, however, demonstrated a shift in these scores, suggesting that the therapeutic intervention had a measurable impact on the tumor’s immunological profile. These findings underscore the dynamic nature of tumor-immune interactions and support the inclusion of both CPS and TPS as biomarkers in assessing response to therapy. The significant difference in progression-free survival (PFS) between CPS-positive and CPS-negative subjects, but not between TPS-positive and TPS-negative subjects, further highlights the potential of CPS as a prognostic marker for therapeutic outcomes in HCC.

## 4. Discussion

In light of recent advancements, Immuno-Oncology (IO) combination therapies have emerged as transformative in the first-line management of Hepatocellular Carcinoma (HCC) in patients classified within BCLC stage C [15]. Notably, the synergy between atezolizumab, a PD-L1 inhibitor, and bevacizumab, a VEGF inhibitor, has demonstrated promising anti-tumor efficacy and an acceptable safety profile in a phase 1b trial encompassing individuals with unresectable HCC. These findings were further substantiated by the pivotal IMbrave-150 trial, which established the atezolizumab-bevacizumab duo as the new standard in the initial treatment regimen for HCC within this specific staging, evidencing a superior median overall survival (OS) compared to sorafenib (19.2 vs. 13.4 months; *p* < 0.001), thereby garnering recommendation across several international treatment guidelines [22].

Additional investigations have explored various combinations to enhance treatment efficacy further. The HIMALAYA trial explored the integration of tremelimumab, a CTLA-4 antagonist, with durvalumab, a PD-L1 antagonist, revealing a significant uplift in median OS against sorafenib in a first-line setting, although awaiting FDA and EMA approval. Concurrently, the CheckMate 9DW study evaluates the nivolumab and ipilimumab combination, with outcomes pending [23].

Contrastingly, the LEAP-002 trial, assessing the combination of pembrolizumab and lenvatinib versus lenvatinib alone, did not meet its primary endpoints, highlighting the complex nature of advancing HCC treatment [24]. However, a notable study, COSMIC-312, investigated cabozantinib in combination with atezolizumab versus sorafenib, demonstrating a progression-free survival benefit despite not reaching statistical significance in median OS [25].

These advancements underscore the potential of IO combination therapies in altering the treatment landscape for HCC. However, the variability in outcomes and ongoing trials illustrate the need for further research to delineate the optimal use of these combinations in clinical practice fully.

Our study acknowledges the critical role of nodule biopsy in providing insightful data post-local interventional procedures, potentially influencing prognosis and therapeutic direction. Despite advancements in imaging techniques diminishing the need for biopsy in HCC diagnosis, the procedure remains pivotal in certain contexts, notably for characterizing tumor biology and guiding therapeutic decisions in the era of emerging systemic therapies. This approach is particularly relevant given the heterogeneity of HCC and the evolving landscape of treatment options, where novel therapies like IO combinations are becoming increasingly integral.

As we navigate the complexities of HCC management, our research highlights the importance of continuous investigation into the efficacy of neoadjuvant immune therapies and the value of histological analysis in tailoring patient-specific treatment strategies. Amidst the challenges of limited sample sizes and the retrospective nature of studies, our findings advocate for a nuanced understanding of HCC’s biological underpinnings to enhance treatment outcomes and patient survival in this diverse patient population [26].

With the advent of sophisticated imaging technologies, the necessity for liver biopsy in establishing HCC diagnoses has diminished. Given the prevalence of HCC among individuals with cirrhosis, who often present with advanced disease and coagulopathy, the procedural risk profile, including severe hemorrhage, abscess formation, and portal vein thrombosis, becomes markedly elevated. A recent meta-analysis highlighted that bleeding incidents of any magnitude were observed in 10.9% of image-guided liver biopsies [27]. Specifically, for HCC biopsies, there is a notable risk of tumor cell dissemination along the needle path, reported between 1.5% and 5.8% of instances. Nevertheless, more contemporary retrospective analyses have indicated a minimal risk of 0.13% for tumor seeding solely from liver biopsies, with an increased risk of 1.82% following ablation procedures [28].

There is a strong point to be made in favor of avoiding liver biopsy for HCC diagnosis. Imaging techniques such as contrast-enhanced ultrasonography (CEUS) and CT scan have high sensitivity and specificity for HCC (64.1% sensitivity, 97.4% specificity, and 73.6% accuracy for CEUS, 62.35% sensitivity, 73.85% specificity, and 67.33% accuracy for CT scan) [29,30]. In addition to this, magnetic resonance imaging, particularly using contrast, is more specific for the detection of small HCC lesions [31,32]. Combining the imaging aspects with serum biomarkers such as AFP in a patient with a diagnosed liver disease increases diagnostic accuracy. Histology diagnosis may be required in the setting of patients without cirrhosis [5]. Liver biopsy has a varied sensitivity in several studies (ranging from 66% to 93%, depending on tumor and needle size as well as operator experience), with a 100% specificity and positive predictive value [33]. On the other hand, even the LIRADS classification of liver nodules reserves certain cases to be diagnosed by biopsy [11]. This is the case of LR-4 nodules (with a 74% risk of HCC and an 80% risk of malignancy) and LR-TIV (the presence of malignant portal vein thrombus with or without liver nodule). In our study, all LR-4 cases were histologically proven, confirming the imaging diagnosis.

The main advantage of liver biopsy is providing useful markers for prognosis and therapeutic schemes, in the current setting of interventional and systemic options. To this end, one of the most studied markers was the expression of PD-L1 in tumor cells, explored in validation studies for ICI. PD-L1 positive tumors have a better response to nivolumab or pembrolizumab compared to sorafenib [34,35]. However, the overall response rates were lower than expected, regardless of PD-L1 expression. It appears that PD-L1 expression in immune cells infiltrating the tumor better correlates to the response to ICI in several solid neoplasia [36]. A recent review argues in favor of using PD-L1 expression on immune cells rather than tumor cells as a prognosis marker for the response to anti-PD-1/PD-L1 response [37].

In our study, we found an expression of PD-L1 on tumor cells varying between 8.7% and 11.6%, similar to literature data [38]. We did not evaluate the expression of PD-1/PD-L1 in immune cells. This particular line of research is under development, as there are uncertainties regarding the histochemical methods of evaluating this expression, cut-off values, and what parameters are better associated with patient prognosis. These uncertainties arise from the different methods and antibodies used to determine PD-L1 expression, as this is not yet standardized [39]. Also, PD-L1 expression in tumor cells changes over time, and this has not yet been evaluated as a prognostic factor [40]. In addition to this, there are two possible methods of defining PD-L1 positive expression: the proportion of PD-L1 positive tumor cells and the ratio of PD-L1 positive tumor cells and immune cells. The latter appears to be a more reliable marker for prognosis [35].

As an expression of ongoing research in HCC therapies is the high number of clinical trials studying the efficacy of neoadjuvant immune therapy [41,42]. For example, the use of camrelizumab (PD-1 inhibitor) and apatinib (tyrosine kinase inhibitor) in treatment-naïve patients with resectable HCCs led to a major pathologic response in nearly a third of patients [43].

Little data exist on performing tumor biopsy after a local interventional procedure, as these patients should be monitored by multimodal imaging according to the international consensus [44]. Histology data in these cases are mostly obtained by analyzing the explanted liver after transplantation. An interesting comparison between non-treated HCCs and post-TACE HCCs revealed that TACE induces morphological changes in the tumor cells (producing a hepatocholangiocellular phenotype) as well as in the tumoral microenvironment (active endothelial proliferation adjacent to the necrotic area [45,46,47]. These changes are associated with chemoresistance and a worse outcome. Therefore, the emerging systemic therapies bring to attention the importance of tumor histology for the prognosis of the patients, even in the setting of a clear imaging diagnosis [48,49].

This study is subject to several constraints that warrant mention. Primarily, the limited sample size of 23 patient biopsies restricts the statistical power of our analyses and precludes a robust correlation of pathological observations with HCC’s underlying etiology. The retrospective design of our investigation further limits our capacity to conduct sequential liver biopsies on the same patients before and after local interventional procedures, which might have yielded insights into prognostic histological markers. Additionally, this study was not designed to assess patient outcomes relative to the response to immunotherapy, attributed partly to the restricted accessibility of these treatments within our geographical locale.

The lack of comprehensive data on treatment responses and long-term patient outcomes underpins another significant limitation of our current research, emanating predominantly from the study’s retrospective nature and the abbreviated follow-up period for a subset of participants. We intend to address these shortcomings in future research endeavors, which will aim to systematically evaluate patient outcomes over an extended timeline, encompassing response rates to diverse treatment regimens and overall survival. Such longitudinal analyses are critical for elucidating the effectiveness of various therapeutic strategies in managing HCC.

A notable observation in our study was the general reduction in Alpha-Fetoprotein (AFP) levels following the initial treatment phase. However, the absence of a nuanced analysis distinguishing between AFP-negative and AFP-positive HCC cases marks a significant research gap. Future investigations will seek to delineate the prognostic and therapeutic significance of AFP levels pre- and post-treatment, with a view to refining risk stratification and personalizing treatment paradigms for HCC patients.

Despite these limitations, our study underscores the value of conducting ongoing research in the domain of HCC, particularly emphasizing the potential to glean meaningful histological data post-therapeutic interventions. Our exploration into three distinct treatment scenarios—comprising patients with no prior interventions, those undergoing Transarterial Chemoembolization (TACE), and those subject to Radiofrequency Ablation (RFA)—was driven by an intent to uncover histopathological variances contingent upon treatment history. Although challenged by a constrained sample size, a ubiquitous hurdle in specialized, condition-specific inquiries, our findings advocate for the sustained pursuit of knowledge in HCC research, highlighting the critical role of histological analysis in advancing our understanding and treatment of this complex disease.

We argue that in the setting of limited resources for liver transplantation (a curative therapeutic option for HCC) tumor biopsy and immune histochemical analysis should be performed at any time during patient management, in order to optimize and customize the therapeutic approach. For example, a single center trial showed increased median survival over 3 years in patients with advanced HCC on sequential systemic therapies alone [42,50,51].

## 5. Conclusions

In conclusion, the practice of conducting biopsies on Hepatocellular Carcinoma (HCC) lesions, even subsequent to local interventional treatments, can be instrumental in unveiling critical data that significantly influence the determination of the most effective treatment strategies for patients. This approach not only has the potential to reveal the HCC phenotype with greater clarity but also to provide insights into the tumor’s behavior and response to previous treatments. Such information is invaluable in tailoring personalized therapeutic plans that are more likely to result in successful outcomes.

As the landscape of HCC management evolves with the introduction and integration of new systemic therapies, there is a pressing need to revisit and refine existing protocols for sequencing these treatments. Biopsies, particularly those performed in high-caliber tertiary care centers, are pivotal in this process. They not only contribute to a deeper understanding of the disease at the molecular and cellular levels but also ensure that the data collected can support ongoing clinical trials and systematic reviews. This collaborative and systematic approach to data collection and analysis is essential for advancing the field of HCC treatment.

Moreover, the role of biopsies in identifying the phenotypic characteristics of HCC after local procedures cannot be overstated. By providing a window into the tumor’s morphological and molecular changes post-treatment, biopsies can guide the selection of subsequent interventions, including the possibility of incorporating emerging systemic therapies that may be more effective against specific tumor phenotypes or in the context of the tumor’s altered microenvironment.

In light of these considerations, it is imperative that the medical community, particularly those specializing in oncology and hepatology, recognize the value of biopsies not just as a diagnostic tool but as a cornerstone of comprehensive patient care in HCC. This entails not only the execution of biopsies with precision and safety but also the integration of biopsy findings into a broader, multidisciplinary discussion on patient management. Through this, clinicians can ensure that each patient’s treatment plan is as informed, nuanced, and effective as possible, ultimately leading to better prognostic outcomes and enhanced quality of life for individuals afflicted with this challenging and complex disease.

## Figures and Tables

**Table 1:** Demographic and imaging data.

	No Previous Interventions (N = 10 Patients)	Previous TACE (N = 9 Patients)	Previous RFA (N = 4 Patients)
Etiology of liver disease	HCV cirrhosis: 2 patients (20%) HBV cirrhosis: 5 patients (50%) NASH cirrhosis: 1 patient (10%) HBV + HDV hepatitis: 2 patients (20%)	HCV cirrhosis: 5 patients (55.5%) HBV hepatitis: 1 patient (11.1%) HBV + HDV hepatitis: 2 patients (22.2%) NASH cirrhosis: 1 patient (11.1%)	HCV cirrhosis: 2 patients (50%) HBV hepatitis: 1 patient (25%) HBV cirrhosis: 1 patient (25%)
AFP (N: 0–8.1 ng/mL)	380.52 ± 134.83	112.56 ± 45.24	135 ± 46.13
LI-RADS score	LR-4: 2 patients (20%) LR-5: 8 patients (80%)	LR-4: 2 patients (22.2%) LR-5: 7 patients (77.7%)	LR-4: 1 patient (25%) LR-5: 3 patients (75%)
Number of nodules	1–3: 8 patients (80%) >3: 2 patients (20%)	1–3: 6 patients (66.6%) >3: 3 patients (33.3%)	1–3: 3 patients (75%) >3: 1 patient (25%)
Size of nodule of interest	Median 3.5 cm Range 1.6–5.2 cm	Median 2.4 cm Range 1.8–3.7	Median 2.2 cm Range 2.1–2.5 cm
Portal vein invasion	Yes—4 patients (40%)	Yes—2 patients (22.2%)	Yes—0 patients (0%)

TACE: transarterial chemoembolization, RFA: radiofrequency ablation, AFP: alpha-fetoprotein, HCV hepatitis C virus. HBV hepatitis B virus, HDV: hepatitis D virus, NASH non-alcoholic steatohepatitis.

**Table 2:** Histology and immunohistochemistry analysis of biopsies.

	No Previous Interventions (N = 10 Patients)	Previous TACE (N = 9 Patients)	Previous RFA (N = 4 Patients)
Edmondson–Steiner	Grade II: 4 patients (40%)	Grade II: 3 patients (33.3%)	Grade II: 1 patient (25%)
	Grade III: 5 patients (50%)	Grade III: 6 patients (66.6%)	Grade III: 3 patients (75%)
	Grade IV: 1 patient (1%)		
PD-L1 expression	11.6%	8.7%	9.4%

TACE, transarterial chemoembolization; RFA, radiofrequency ablation; PD-L1, programmed cell death ligand 1.

## Data Availability

Materials and raw data may be given upon request to the corresponding author.

## References

[1] Lee H.M., Lidofsky S.D., Taddei T.H., Townshend-Bulson L.J. (2023). Attacking the public health crisis of hepatocellular carcinoma at its roots. Hepatology.

[2] Bray F., Ferlay J., Soerjomataram I., Siegel R.L., Torre L.A., Jemal A. (2018). Global cancer statistics 2018: GLOBOCAN estimates of incidence and mortality worldwide for 36 cancers in 185 countries. CA Cancer J. Clin..

[3] Rumgay H., Arnold M., Ferlay J., Lesi O., Cabasag C.J., Vignat J., Laversanne M., McGlynn K.A., Soerjomataram I. (2022). Global burden of primary liver cancer in 2020 and predictions to 2040. J. Hepatol..

[4] Forner A., Reig M., Bruix J. (2018). Hepatocellular carcinoma. Lancet.

[5] European Association for the Study of the Liver (2018). European Association for the Study of the Liver. EASL Clinical Practice Guidelines: Management of hepatocellular carcinoma. J. Hepatol..

[6] Valery P.C., Laversanne M., Clark P.J., Petrick J.L., McGlynn K.A., Bray F. (2018). Projections of primary liver cancer to 2030 in 30 countries worldwide. Hepatology.

[7] Singal A.G., Llovet J.M., Yarchoan M., Mehta N., Heimbach J.K., Dawson L.A., Jou J.H., Kulik L.M., Agopian V.G., Marrero J.A. (2023). AASLD Practice Guidance on prevention, diagnosis, and treatment of hepatocellular carcinoma. Hepatology.

[8] Eisenbrey J.R., Gabriel H., Savsani E., Lyshchik A. (2021). Contrast-enhanced ultrasound (CEUS) in HCC diagnosis and assessment of tumor response to locoregional therapies. Abdom. Radiol..

[9] Matsui O., Kobayashi S., Sanada J., Kouda W., Ryu Y., Kozaka K., Kitao A., Nakamura K., Gabata T. (2011). Hepatocelluar nodules in liver cirrhosis: Hemodynamic evaluation (angiography-assisted CT) with special reference to multi-step hepatocarcinogenesis. Abdom. Imaging.

[10] Huang P., Wu F., Hou K., Zhou C., Xiao Y., Wang C., Miao G., Yang C., Zeng M. (2023). Diagnostic algorithm for subcentimeter hepatocellular carcinoma using alpha-fetoprotein and imaging features on gadoxetic acid-enhanced MRI. Eur. Radiol..

[11] Lee Y.T., Wang J.J., Zhu Y., Agopian V.G., Tseng H.R., Yang J.D. (2021). Diagnostic Criteria and LI-RADS for Hepatocellular Carcinoma. Clin. Liver Dis..

[12] Chernyak V., Fowler K.J., Kamaya A., Kielar A.Z., Elsayes K.M., Bashir M.R., Kono Y., Do R.K., Mitchell D.G., Singal A.G. (2018). Liver Imaging Reporting and Data System (LI-RADS) Version 2018: Imaging of Hepatocellular Carcinoma in At-Risk Patients. Radiology.

[13] Tremosini S., Forner A., Boix L., Vilana R., Bianchi L., Reig M., Rimola J., Rodríguez-Lope C., Ayuso C., Solé M. (2012). Prospective validation of an immunohistochemical panel (glypican 3, heat shock protein 70 and glutamine synthetase) in liver biopsies for diagnosis of very early hepatocellular carcinoma. Gut.

[14] Park B.V., Gaba R.C., Huang Y.H., Chen Y.F., Guzman G., Lokken R.P. (2019). Histology of Hepatocellular Carcinoma: Association with Clinical Features, Radiological Findings, and Locoregional Therapy Outcomes. J. Clin. Imaging Sci..

[15] Reig M., Forner A., Rimola J., Ferrer-Fàbrega J., Burrel M., Garcia-Criado Á., Kelley R.K., Galle P.R., Mazzaferro V., Salem R. (2022). BCLC strategy for prognosis prediction and treatment recommendation: The 2022 update. J. Hepatol..

[16] Bruix J., Chan S.L., Galle P.R., Rimassa L., Sangro B. (2021). Systemic treatment of hepatocellular carcinoma: An EASL position paper. J. Hepatol..

[17] He Y., Lu M., Che J., Chu Q., Zhang P., Chen Y. (2021). Biomarkers and Future Perspectives for Hepatocellular Carcinoma Immunotherapy. Front. Oncol..

[18] Yau T., Park J.W., Finn R.S., Cheng A.L., Mathurin P., Edeline J., Kudo M., Harding J.J., Merle P., Rosmorduc O. (2022). Nivolumab versus sorafenib in advanced hepatocellular carcinoma (CheckMate 459): A randomised, multicentre, open-label, phase 3 trial. Lancet Oncol..

[19] Ji J.H., Ha S.Y., Lee D., Sankar K., Koltsova E.K., Abou-Alfa G.K., Yang J.D. (2023). Predictive Biomarkers for Immune-Checkpoint Inhibitor Treatment Response in Patients with Hepatocellular Carcinoma. Int. J. Mol. Sci..

[20] Calderaro J., Rousseau B., Amaddeo G., Mercey M., Charpy C., Costentin C., Luciani A., Zafrani E.S., Laurent A., Azoulay D. (2016). Programmed death ligand 1 expression in hepatocellular carcinoma: Relationship with clinical and pathological features. Hepatology.

[21] Cheng A.L., Qin S., Ikeda M., Galle P.R., Ducreux M., Kim T.Y., Lim H.Y., Kudo M., Breder V., Merle P. (2022). Updated efficacy and safety data from IMbrave150: Atezolizumab plus bevacizumab vs. sorafenib for unresectable hepatocellular carcinoma. J. Hepatol..

[22] Lee M.S., Ryoo B.-Y., Hsu C.-H., Numata K., Stein S., Verret W., Hack S.P., Spahn J., Liu B., Abdullah H. (2020). Atezolizumab with or without Bevacizumab in Unresectable Hepatocellular Carcinoma (GO30140): An Open-Label, Multicentre, Phase 1b Study. Lancet Oncol..

[23] Finn R.S., Qin S., Ikeda M., Galle P.R., Ducreux M., Kim T.-Y., Kudo M., Breder V., Merle P., Kaseb A.O. (2020). Atezolizumab plus Bevacizumab in Unresectable Hepatocellular Carcinoma. N. Engl. J. Med..

[24] Kudo M. (2022). Durvalumab plus Tremelimumab in Unresectable Hepatocellular Carcinoma. Hepatobiliary Surg. Nutr..

[25] Nawaz K. (2022). ESMO 2022: Ten Key Takeaways on Europe’s Top Oncology Event.

[26] Formular Pentru Verificarea Respectării Criteriilor de Eligibilitate Aferente Protocolului Terapeutic Dci Atezolizumab. http://www.casan.ro/casbz/media/pageFiles/201)%20L01XC32.5-ATEZOLIZUMAB%20carcinom%20hepatocelular.pdf.

[27] Chiang C.L., Chan A.C.Y., Chiu K.W.H., Kong F.S. (2019). Combined Stereotactic Body Radiotherapy and Checkpoint Inhibition in Unresectable Hepatocellular Carcinoma: A Potential Synergistic Treatment Strategy. Front. Oncol..

[28] Finn R.S. (2016). The Role of Liver Biopsy in Hepatocellular Carcinoma. Gastroenterol. Hepatol..

[29] Seeff L.B., Everson G.T., Morgan T.R., Curto T.M., Lee W.M., Ghany M.G., Shiffman M.L., Fontana R.J., Di Bisceglie A.M., Bonkovsky H.L. (2010). Complication rate of percutaneous liver biopsies among persons with advanced chronic liver disease in the HALT-C trial. Clin. Gastroenterol. Hepatol..

[30] Midia M., Odedra D., Shuster A., Midia R., Muir J. (2019). Predictors of bleeding complications following percutaneous image-guided liver biopsy: A scoping review. Diagn. Interv. Radiol..

[31] Silva M.A., Hegab B., Hyde C., Guo B., Buckels J.A., Mirza D.F. (2008). Needle track seeding following biopsy of liver lesions in the diagnosis of hepatocellular cancer: A systematic review and meta-analysis. Gut.

[32] Szpakowski J.L., Drasin T.E., Lyon L.L. (2017). Rate of seeding with biopsies and ablations of hepatocellular carcinoma: A retrospective cohort study. Hepatol. Commun..

[33] Liu G.J., Wang W., Lu M.D., Xie X.Y., Xu H.X., Xu Z.F., Chen L.D., Wang Z., Liang J.Y., Huang Y. (2015). Contrast-Enhanced Ultrasound for the Characterization of Hepatocellular Carcinoma and Intrahepatic Cholangiocarcinoma. Liver Cancer.

[34] Wang G., Zhu S., Li X. (2019). Comparison of values of CT and MRI imaging in the diagnosis of hepatocellular carcinoma and analysis of prognostic factors. Oncol. Lett..

[35] Semaan S., Vietti Violi N., Lewis S., Chatterji M., Song C., Besa C., Babb J.S., Fiel M.I., Schwartz M., Thung S. (2020). Hepatocellular carcinoma detection in liver cirrhosis: Diagnostic performance of contrast-enhanced CT vs. MRI with extracellular contrast vs. gadoxetic acid. Eur. Radiol..

[36] Peng J., Zheng J., Yang C., Wang R., Zhou Y., Tao Y.Y., Gong X.Q., Wang W.C., Zhang X.M., Yang L. (2020). Intravoxel incoherent motion diffusion-weighted imaging to differentiate hepatocellular carcinoma from intrahepatic cholangiocarcinoma. Sci. Rep..

[37] Di Tommaso L., Spadaccini M., Donadon M., Personeni N., Elamin A., Aghemo A., Lleo A. (2019). Role of liver biopsy in hepatocellular carcinoma. World J. Gastroenterol..

[38] Li Y., Liang X., Li H., Yang T., Guo S., Chen X. (2022). Nivolumab Versus Sorafenib as First-Line Therapy for Advanced Hepatocellular Carcinoma: A Cost-Effectiveness Analysis. Front Pharmacol..

[39] Zhu A.X., Finn R.S., Edeline J., Cattan S., Ogasawara S., Palmer D., Verslype C., Zagonel V., Fartoux L., Vogel A. (2018). Pembrolizumab in patients with advanced hepatocellular carcinoma previously treated with sorafenib (KEYNOTE-224): A non-randomised, open-label phase 2 trial. Lancet Oncol..

[40] Powles T., Eder J.P., Fine G.D., Braiteh F.S., Loriot Y., Cruz C., Bellmunt J., Burris H.A., Petrylak D.P., Teng S.L. (2014). MPDL3280A (anti-PD-L1) treatment leads to clinical activity in metastatic bladder cancer. Nature.

[41] Kleinovink J.W., van Hall T., Ossendorp F., Fransen M.F. (2017). PD-L1 immune suppression in cancer: Tumor cells or host cells?. Oncoimmunology.

[42] Von Felden J., Karkmann K., Ittrich H., Gil-Ibanez I., Fründt T., Krause J., Lohse A.W., Wege H., Schulze K. (2021). Sequential Systemic Treatment in Advanced Hepatocellular Carcinoma Is Able to Prolong Median Survival to More than 3 Years in a Selected Real-World Cohort. Visc. Med..

[43] Pinato D.J., Mauri F.A., Spina P., Cain O., Siddique A., Goldin R., Victor S., Pizio C., Akarca A.U., Boldorini R.L. (2019). Clinical implications of heterogeneity in PD-L1 immunohistochemical detection in hepatocellular carcinoma: The Blueprint-HCC study. Br. J. Cancer.

[44] Yi M., Jiao D., Xu H., Liu Q., Zhao W., Han X., Wu K. (2018). Biomarkers for predicting efficacy of PD-1/PD-L1 inhibitors. Mol. Cancer.

[45] Vilain R.E., Menzies A.M., Wilmott J.S., Kakavand H., Madore J., Guminski A., Liniker E., Kong B.Y., Cooper A.J., Howle J.R. (2017). Dynamic Changes in PD-L1 Expression and Immune Infiltrates Early During Treatment Predict Response to PD-1 Blockade in Melanoma. Clin. Cancer Res..

[46] Zen C., Zen Y., Mitry R.R., Corbeil D., Karbanová J., O’Grady J., Karani J., Kane P., Heaton N., Portmann B.C. (2011). Mixed phenotype hepatocellular carcinoma after transarterial chemoembolization and liver transplantation. Liver Transpl..

[47] Russo F.P., Imondi A., Lynch E.N., Farinati F. (2018). When and how should we perform a biopsy for HCC in patients with liver cirrhosis in 2018? A review. Dig. Liver Dis..

[48] Rallis K.S., Makrakis D., Ziogas I.A., Tsoulfas G. (2022). Immunotherapy for advanced hepatocellular carcinoma: From clinical trials to real-world data and future advances. World J. Clin. Oncol..

[49] Xia Y., Tang W., Qian X., Li X., Cheng F., Wang K., Zhang F., Zhang C., Li D., Song J. (2022). Efficacy and safety of camrelizumab plus apatinib during the perioperative period in resectable hepatocellular carcinoma: A single-arm, open label, phase II clinical trial. J. Immunother. Cancer.

[50] Maas M., Beets-Tan R., Gaubert J.Y., Gomez Munoz F., Habert P., Klompenhouwer L.G., Vilares Morgado P., Schaefer N., Cornelis F.H., Solomon S.B. (2020). Follow-up after radiological intervention in oncology: ECIO-ESOI evidence and consensus-based recommendations for clinical practice. Insights Imaging.

[51] Ciuhu A.N., Rahnea-Nita G., Popescu M., Rahnea-Nita R.A. (2015). Abstract P5–15–22: Evaluation of quality of life in patients with advanced and metastatic breast cancer proposed for palliative chemotherapy and best supportive care versus best supportive care. Cancer Res..

